# α2,3‐Sialyltransferase (ST3Gal1) regulates endometrioid‐type epithelial ovarian cancer cell migration and invasion via VEGF‐R2/JAK2/STAT3 signaling cascades

**DOI:** 10.1002/ijgo.71057

**Published:** 2026-05-04

**Authors:** Wei‐Ting Chao, Chia‐Hao Liu, Szu‐Ting Yang, Chen‐Hao Lin, Liang‐Wei Wang, Peng‐Hui Wang

**Affiliations:** ^1^ Department of Obstetrics and Gynecology Taipei Veterans General Hospital Taipei Taiwan; ^2^ Department of Obstetrics and Gynecology National Yang Ming Chiao Tung University Taipei Taiwan; ^3^ Institute of Clinical Medicine National Yang Ming Chiao Tung University Taipei Taiwan; ^4^ Taiwan Female Cancer Foundation Taipei Taiwan; ^5^ Department of Obstetrics and Gynecology, Faculty of Medicine Universitas Airlangga Surabaya Indonesia; ^6^ Department of Medical Research China Medical University Hospital Taichung Taiwan

**Keywords:** angiogenesis, endometrioid‐type epithelial ovarian cancer, ST3Gal1, VEGF‐R2/JAK2/STAT3 signaling, α2,3‐sialyltransferase

## Abstract

**Objective:**

To investigate the role of ST3 β‐galactoside α‐2,3‐sialyltransferase 1 (ST3Gal1) and vascular endothelial growth factor receptor 2 (VEGF‐R2) in endometrioid‐type epithelial ovarian cancer (E‐OC) because aberrant α2,3‐sialylation mediated by ST3Gal1 and VEGF‐R2‐related angiogenesis is linked with tumor progression.

**Methods:**

ST3Gal1 and VEGF‐R2 expression levels were analyzed in E‐OC tissues and cell lines. ST3Gal1 knockdown and ST3Gal1 inhibitor soyasaponin I (SsaI) treatment were performed to evaluate the effects of altered sialylation on the VEGF‐R2 pathway, downstream Janus kinase 2/signal transducer and activator of transcription 3 (JAK2/STAT3) signaling, and cell migration/invasion. Co‐immunoprecipitation experiments were conducted to confirm the interaction between ST3Gal1 and VEGF‐R2. The combination of SsaI and VEGF‐R2 inhibitors was assessed using in vitro and in vivo studies.

**Results:**

High ST3Gal1 expression was associated with advanced E‐OC stage and poorer overall survival in a univariable analysis; however, it did not retain independent prognostic significance in a multivariable analysis. ST3Gal1 knockdown reduced VEGF‐R2 expression and inhibited downstream JAK2/STAT3 signaling and suppressed tumor cell migration and invasion. SsaI treatment reduced VEGF‐R2 signaling and impaired metastatic potential in vitro. The combined inhibition of ST3Gal1 and VEGF‐R2 reduced tumor growth in vivo.

**Conclusions:**

Targeting ST3Gal1‐mediated α2,3‐sialylation disrupts VEGF‐R2 signaling and suppresses metastatic behavior in E‐OC models. Although clinical associations suggest a potential link with adverse outcomes, larger studies are required to clarify its independent prognostic significance. Combined inhibition of ST3Gal1 and VEGF‐R2 reduced tumor growth in vivo; these findings support further investigations of ST3Gal1 as a potential therapeutic target in E‐OC.

## 1 INTRODUCTION

Epithelial ovarian cancer (OC), the second most common gynecologic cancer, is a leading cause of cancer‐related deaths worldwide.^1^, ^2^, ^3^ Despite intensive treatment with standard‐of‐care therapy including maximal cytoreductive surgery (CRS) plus perioperative (neoadjuvant and/or postoperative adjuvant) chemotherapy with or without anti‐angiogenesis agent (bevacizumab) maintenance therapy, the outcome is poor, particularly in cases of advanced‐stage OC (ads‐OC).^3^, ^4^, ^5^ Biomarker‐driven therapy based on genomic evaluations has recently become one of the most important strategies for treating patients with ads‐OC.^6^, ^7^ For example, the use of a poly(ADP‐ribose) polymerase (PARP) inhibitor (e.g., olaparib) with or without bevacizumab as maintenance therapy for treating patients with ads‐OC and homologous recombination deficiency‐positive tumors (a phenotype of defective homologous recombination repair pathway, regardless of *BRCA* mutation [*BRCA*m]) status, both prolongs progression‐free survival (PFS) and possibly increases the overall survival (OS) rate.^8^, ^9^

In contrast, for treating patients with ads‐OC and a homologous recombination proficiency, the National Comprehensive Cancer Network® guideline still recommends paclitaxel‐carboplatin with bevacizumab maintenance therapy as a preferred regimen,^9^ suggesting that an anti‐angiogenesis agent is still an essential backbone for nearly all ads‐OC cases.^2^ Therefore, enhancing the therapeutic effect of, or overcoming resistance to, anti‐angiogenesis agents may be an alternative strategy that can improve the outcomes of patients with cancer,^10^ including those with OC.^11^

Our previous studies demonstrated that β‐galactoside α‐2,3‐sialyltransferase 1 (ST3Gal1)‐involved α2,3‐linked sialylation was significantly increased in both high‐grade serous and clear‐cell OC.^12^, ^13^ However, the role of altered sialylation in endometrioid‐type OC (E‐OC) is unknown. We also determined that targeting ST3Gal1 can overcome anti‐vascular endothelial growth factor (VEGF) resistance in endometrial cancer (EC).^14^ As E‐OC shows an endometrioid histology (similar to endometrioid EC) and is associated with endometriosis (similar to clear‐cell OC as endometriosis‐associated OC),^15^ the current study aimed to investigate the relationship between ST3Gal1 and VEGF receptor 2 (VEGF‐R2) as well as their role in patients with E‐OC.

## 2 MATERIALS AND METHODS

### 2.1 Patients and tissue samples

This study represents a retrospective translational cohort study conducted at Taipei Veterans General Hospital. E‐OC tissues collected from 53 patients who underwent CRS at Taipei Veterans General Hospital since January 2000 were collected with institutional review board approval (no. 2021–02‐001B). Samples were snap‐frozen and stored at −80°C. Written informed consent was obtained from all patients for the use of their tumor specimens for research purposes.

Patients with pathologically confirmed E‐OC who underwent CRS and for whom tumor tissues were available for the research analysis were included. Patients for whom no tumor tissue samples were available or for whom clinicopathologic data were insufficient were excluded.

Residual disease was categorized according to the largest‐diameter visible tumor remaining after maximal CRS.^4^, ^5^, ^16^ R0 was defined as no macroscopic residual disease, R1 was defined as a residual tumor up to 1 cm in greatest diameter, and R2 indicated a residual tumor of greater than 1 cm after CRS.^16^ Early‐stage OC (eas‐OC) was defined as FIGO (the International Federation of Gynecology & Obstetrics) Stage I–II, whereas ads‐OC was defined as FIGO Stage III–IV according to the FIGO OC classification system.^3^ Recurrence was defined as radiologic evidence of disease progression after the completion of primary treatment. Platinum‐free interval was defined as the interval between the completion of platinum‐based chemotherapy and the first documented recurrence. Platinum sensitivity was defined according to platinum‐free interval, with recurrence occurring at 6 months or later considered platinum‐sensitive OC and that occurring after less than 6 months considered platinum‐resistant OC.^11^ OS was defined as the interval from primary treatment to death or the date of last follow up (through December 30, 2025).

### 2.2 Cell culture

For human E‐OC cell lines, A2780 was purchased from Sigma‐Aldrich and TOV‐112D was purchased from the Bioresource Collection and Research Center (Taiwan). To ensure cell‐line identity, short tandem repeat (STR) profiling was performed and the results were compared with reference databases. The STR authentication confirmed a 100% match with the reference A2780 and TOV‐112D profiles. Moreover, cell‐line information and histologic annotations were verified using the Cellosaurus cell‐line information database,^17^ a comprehensive cell‐line knowledge resource that integrates data from major cell repositories including the American Type Culture Collection, the Deutsche Sammlung von Mikrorganismen and Zellkulturen, and the Japanese Collection of Research Bioresources Cell Bank.^17^ According to the Cellosaurus database, TOV‐112D is derived from E‐OC, and A2780 has been widely used as a model of OC with a reported E‐OC origin. The STR authentication reports are provided in Appendix S1.

A2780/A2780R and TOV112D/TOV112DR cell lines were maintained in RPMI‐1640 medium (Gibco) or in a 1:1 mixture of MCDB131 and Medium 199 (Gibco) with 10%–15% fetal bovine serum and 1% penicillin–streptomycin at 37°C in a humidified atmosphere with 5% CO_2_. Cisplatin‐resistant cell lines were cultured with 2 μM cisplatin. Cell‐line identity was verified through STR analysis.

### 2.3 Animal experiments

Twenty‐four 6‐week‐old female BALB/c athymic (nu+/nu+) mice were injected subcutaneously into the flanks with 6 × 10^6^ A2780R cells in 200 μL of phosphate‐buffered saline/Matrigel (1:1; Collaborative Biomedical Products). When tumors reached approximately 100 mm^3^, the mice were randomly assigned by a computer‐generated randomization schedule into control, soyasaponin I (SsaI; 20 mg/kg), cabozantinib (XL184; 30 mg/kg), or combination therapy groups (*n* = 6 per group). Oral treatments were administered daily for 14 days, with tumor volume and body weight measured every 3 days by investigators blinded to the treatment allocations to minimize measurement bias. Tumors were harvested for analysis. All procedures were approved by the Institutional Animal Care and Use Committee (protocol no. 2020–285).

### 2.4 ST3Gal1 gene overexpression and knockdown

Lentiviral short hairpin RNA vectors targeting ST3Gal1 (KD#1: 5′‐GCGGGAGAAGAAGCCCAATAA‐3′; KD#2: 5′‐AGATAGACAGTCACGACTTTG‐3′) and control vectors provided by RNAi Core Facility (Academia Sinica, Taiwan) were transduced into OC cells using CaCl_2_ and polybrene following the manufacturer's instructions. Stable lines were selected with puromycin (1 μg/mL; Sigma‐Aldrich). For overexpression, cells were transfected with ST3Gal1 expression plasmids (Origene) or empty vectors (SignaGenigma).

### 2.5 Western blotting analysis

Cells were lysed in RIPA buffer (Sigma‐Aldrich). After 30 min on ice with vortexing, the lysates were centrifuged (16,000 x *g*, 30 min, 4°C) to remove debris. A total of 50 μg of protein was mixed with 4× sample buffer (Bio‐Rad) containing 125 mM Tris–HCl (pH 6.8), 4% sodium dodecyl sulfate (SDS), 0.02% bromophenol blue, 0.2 M dithiothreitol, and 20% glycerol. Proteins were resolved by 6%, 10%, or 15% SDS‐polyacrylamide gel electrophoresis (SDS‐PAGE) at 100 V for 3 h and transferred to Immobilon polyvinylidene fluoride membranes (Millipore) at 90 V for 2 h. The membranes were blocked in 5% nonfat dry milk/0.1% Tween‐20/Tris‐buffered saline for 30 min at room temperature, incubated overnight at 4°C with primary antibodies (1:3000), then washed and incubated with horseradish peroxidase‐conjugated secondary antibodies (1:3000) for 2 h. The blots were developed using electrochemiluminescence reagent (Bio‐Helix, Taiwan) and visualized with UVP ChemStudio Touch (Level, Taiwan). Densitometry was performed using ImageJ software. Band intensities were normalized to the corresponding loading control (GAPDH, glyceraldehyde 3‐phosphate dehydrogenase) and are expressed relative to the control group. All Western blotting experiments were independently repeated at least three times. The densitometric values shown represent results from three independent biologic experiments.

### 2.6 Immunoprecipitation/co‐immunoprecipitation

Pierce™ immunoprecipitation/co‐immunoprecipitation kits (#88805; Thermo Fisher Scientific) were used. The cells were lysed in Nonidet P‐40 lysis buffer (0.025 M Tris–HCl, 0.15 M NaCl, 1 mM ethylenediaminetetraacetic acid, 1% Nonidet P‐40, and 5% glycerol) with protease inhibitor (Roche) for 1 h. After the centrifugation, the supernatant protein concentration was measured and 1 mg of protein was incubated overnight with cross‐linked antibody beads. Beads incubated without antibody (beads‐only condition) were included as a negative control for the evaluation of nonspecific protein binding to the beads. The immunoprecipitates were washed with 200 μL of immunoprecipitation Lysis/Wash Buffer and eluted with 60 μL of elution buffer (primary amine‐containing). The eluates were resolved with 5× non‐reducing lane marker buffer on 10% SDS‐PAGE gels. A Western blotting analysis was performed to detect VEGF‐R2, ST3Gal1, or phospho‐VEGF‐R2. A lectin affinity immunoprecipitation analysis was performed as previously described.^14^

### 2.7 RNA isolation and real‐time quantitative polymerase chain reaction

RNA was extracted using NucleoZol (MACHEREY‐NAGEL GmbH & Co. KG) and reverse‐transcribed into cDNA using a PrimeScript kit (Takara Bio USA, Inc.,) according to the manufacturer's instructions. Reverse transcription quantitative polymerase chain reaction (RT‐qPCR) was conducted using a CFX96 system (Bio‐Rad) with gene‐specific primers (Roche Assay Design) and 2X SYBR qPCR Mix (BIOTOOLS, Taiwan). Gene expression was normalized to β‐actin.

### 2.8 Immunohistochemistry

Patient tissue sections were subjected to antigen retrieval in a Coplin jar containing retrieval solution (Dako). Sections were then blocked with 1% bovine serum albumin and incubated overnight at 4°C with primary antibody. Horseradish peroxidase‐conjugated secondary antibodies were incubated for 2 h at room temperature. AEC Substrate‐Chromogen (Dako) was used for staining, followed by hematoxylin counterstaining. ST3Gal1 immunohistochemistry staining was evaluated by a pathologist (C‐RL) who was blinded to the clinical data. Staining intensity was scored as 0 (none), 1 (weak), 2 (moderate), or 3 (strong). For the statistical analysis, scores were dichotomized as low expression (0–1) or high expression (2–3) as previously described.^14^ Staining intensity was further quantified using ImageJ software.

### 2.9 Immunofluorescence for DuoLink kit stain

A Duolink® Proximity Ligation Assay (PLA; Olink Bioscience) was used to detect ST3Gal1‐VEGF‐R2 interactions in A2780R cells treated with SsaI or ST3Gal1 knockdown. The cells were fixed, permeabilized, and processed per the manufacturer's instructions. The signals were visualized under a fluorescence microscope.

### 2.10 Cell migration/invasion assay

Transwell assays (8‐μm pores; Corning Inc.) pre‐coated with Matrigel (Corning Inc.) were performed using 2 × 10^4^ cells. Cells were seeded in serum‐free medium in the upper chamber and 10% fetal bovine serum in the lower chamber as a chemoattractant. After 48 h, non‐migrated cells were removed using wet cotton swabs and migrated cells on the underside were fixed, stained with crystal violet, and counted in seven to ten random fields at 200× magnification. Rates were normalized to control using ImageJ.

### 2.11 Colony formation assay

Cells (1500/well) were seeded in six‐well plates and incubated at 37°C for 14 days. Colonies were then fixed, washed, stained with crystal violet, and counted in seven to ten random fields at 200× magnification.

### 2.12 Statistical analysis

Statistical analyses were performed using SPSS v26.0 (IBM, Armonk, NY, USA) and GraphPad Prism 6. Data normality was evaluated using the Shapiro–Wilk test. The *χ* ^2^‐test analyzed categorical variables, while Student *t*‐test or the Wilcoxon rank‐sum test were used to analyze continuous variables. One‐way analysis of variance with Bonferroni correction was used to compare multiple groups. Data are presented as mean ± standard deviation from at least three independent experiments. Univariable and multivariable Cox proportional hazards models were used to assess associations between clinical variables and survival outcomes. The proportional hazards assumption was evaluated by testing time‐dependent covariates in the Cox model. Because this was a retrospective translational cohort study, neither a priori sample size nor a power calculation was performed, and the sample size was determined by the number of eligible patients available during the study period. Statistical significance was defined as a *P*‐value less than 0.05.

## 3 RESULTS

### 3.1 Correlation of ST3Gal1 overexpression with poor prognosis in E‐OC

ST3Gal1 RNA expression was analyzed using Bittner ovarian data from Oncomine, revealing its upregulation in E‐OC (Figure 1a,b). A Kaplan–Meier analysis on the Kaplan–Meier plotter website showed that high ST3Gal1 expression correlated with significantly worse survival in E‐OC.

**FIGURE 1:**
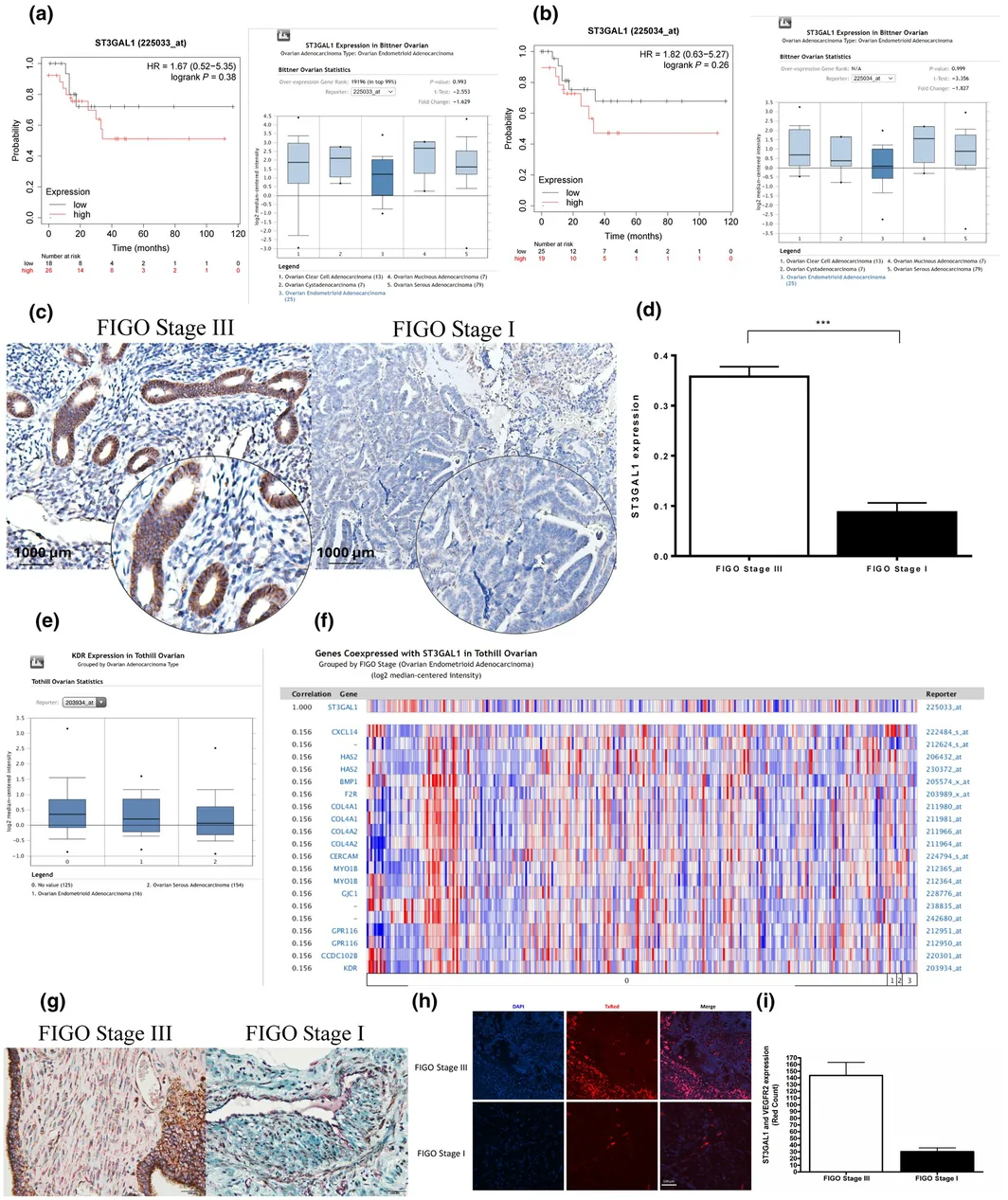
ST3Gal1 overexpression in patients with endometrioid‐type ovarian cancer (E‐OC) is associated with poor prognosis and positively correlates with VEGF‐R2 expression. (a, b) We assessed ST3Gal1 expression in E‐OC tissues from the Bittner dataset on the Oncomine database and performed Kaplan–Meier analysis using the Kaplan–Meier plotter website to evaluate prognosis. (c, d) A comparative immunohistochemical analysis of ST3Gal1 expression in OC tissues of ads‐ and eas‐OC (original magnification: 200×). Data are expressed as mean ± standard deviation (SD); statistical significance was assessed using either analysis of variance or Student's *t*‐test ***P < 0.001. (e, f) Co‐expression analysis using the Oncomine database revealed a positive correlation between ST3Gal1 and VEGF‐R2 in E‐OC. (g) Double staining of ST3Gal1 (pink) and VEGF‐R2 (brown) showed higher expression pattern in the ads‐OC (original magnification: 200×). (h, i) Duolink proximity ligation assay for protein–protein interactions between ST3Gal1 and VEGF‐R2 in E‐OC tissue samples. Each red spot detected represents a single interaction, and the nucleus was stained with DAPI (original magnification: 600×). The red spots were quantified to compare the levels of interaction between ads‐ and eas‐OC samples. Significance was determined using Student's *t*‐test **P < 0.01.

To validate this finding, we used immunohistochemistry to assess ST3Gal1 expression in tissue samples collected from 53 patients with E‐OC (Table 1). ST3Gal1 levels were higher in ads‐E‐OC (Figure 1c,d), with significantly elevated expression in patients with FIGO Stage III/IV disease (*P* < 0.001; Table 1). However, no significant association was found with age, grade, residual disease, tumor size, preoperative cancer antigen 125 (CA‐125) level, or recurrence. Notably, patients with high ST3Gal1 expression had a higher rate of E‐OC‐related death and shorter OS. In univariable analysis, a high ST3Gal1 expression (hazard ratio [HR] 3.63; 95% confidence interval [CI] 1.09–12.10; *P* = 0.036) and advanced FIGO stage (HR 6.07; 95% CI 1.64–22.48; *P* = 0.007) were associated with worse survival (Table 2). However, after the adjustment for clinicopathologic variables, ST3Gal1 expression did not retain statistical significance in the multivariable analysis (HR 1.74; *P* = 0.426). These results suggest that ST3Gal1 may be associated with adverse outcomes in patients with E‐OC; however, due to the absence of statistical significance after the adjustment for clinicopathologic factors, ST3Gal1 expression may not be an independent prognostic factor in the current study.

**TABLE 1:** Analysis of ST3Gal1 expression in endometrioid‐type ovarian cancer specimens.^a^

	Low intensity score (0 ~ 1) (*n* = 32)	High intensity score (2 ~ 3) (*n* = 21)	*P* value
Age at diagnosis, years	50.1 ± 10.5	53.4 ± 13.3	0.310
FIGO stage (%)			<0.001
I	17 (53.1%)^b^	2 (9.6%)	
II	10 (31.3%)	4 (19.0%)	
III & IV	5 (15.6%)	15 (71.4%)^b^	
Histologic grade (%)			0.319
1	4 (12.5%)	1 (4.8%)	
2	20 (62.5%)	11 (52.4%)	
3	8 (25.0%)	9 (42.8%)	
Residual disease (%)			0.069
CCR0	29 (90.6%)	15 (71.4%)	
CCR1	0 (0%)	0 (0%)	
CCR2	3 (9.4%)	6 (28.6%)	
Tumor size, cm	12.0 ± 6.4	10.1 ± 3.1	0.146
CA‐125 (pre‐operation), U/mL	1004 ± 2012	1272 ± 1820	0.625
Recurrence			0.064
Total, *n* (%)	8 (25.0%)	9 (42.9%)	
Platinum‐free interval <6 months	1 (3.1%)	5 (23.8%)	
Platinum‐free interval ≥6 months	7 (21.9%)	4 (19.1%)	
Ovarian cancer death (%)	4 (12.5%)	8 (38.1%)	0.029
Overall survival, months	130.9 ± 86.6	81.3 ± 65.6	0.03

Abbreviations: CCR0, complete resection of tumor without grossly visible lesion; CCR1, remaining small residual tumor burden of 1–10 mm; CCR2, macroscopic residual diseases exceeding 1 cm in diameter; FIGO, the International Federation of Gynecology & Obstetrics.

^a^ Values are presented as mean ± standard deviation or number (percentage).

^b^ Major contributors to the statistical significant difference.

**TABLE 2:** Univariable and multivariable analysis of overall survival predictors by Cox‐regression model.^a^

Variables	Overall survival			
	Univariable		Multivariable	
	HR (95% CI)	*P* value	HR (95% CI)	*P* value
FIGO stage				
I/II	1.00 (reference)		1.00 (reference)	
III/IV	6.07 (1.64–22.48)	0.007	4.60 (1.05–20.15)	0.043
ST3GalI expression				
Low	1.00 (reference)		1.00 (reference)	
High	3.63 (1.09–12.10)	0.036	1.74 (0.45–6.77)	0.426

Abbreviations: CI, confidence interval; FIGO, the International Federation of Gynecology & Obstetrics; HR, hazard ratio; ST3Gal1, ST3 β‐galactoside α‐2,3‐sialyltransferase 1.

^a^ Variables with *P* < 0.05 in the univariable analysis were included in the multivariable model.

### 3.2 Strong co‐expression of ST3Gal1 and VEGF‐R2 in ads‐E‐OC

To explore the underlying mechanism of ST3Gal1 in E‐OC, we performed a co‐expression analysis using the Oncomine Tothill dataset, which revealed VEGF‐R2 (KDR) upregulation in patients with E‐OC (Figure 1e). Our analysis identified a gene cluster co‐expressed with ST3Gal1 in E‐OC including positively correlated VEGF‐R2 (Figure 1f). Double immunohistochemistry staining showed elevated ST3Gal1 (pink) and VEGF‐R2 (brown) expression in ads‐E‐OC (Figure 1g). Duolink PLA, which generated a red signal, further demonstrated increased ST3Gal1‐VEGF‐R2 protein complexes in ads‐E‐OC versus eas‐E‐OC samples (Figure 1h,i), suggesting that their interaction may contribute to E‐OC invasion.

### 3.3 ST3Gal1 knockdown inhibits VEGF‐R2 expression and suppresses cell migration and invasion

We first examined ST3Gal1 and VEGF‐R2 expression in A2780 and TOV112D E‐OC cell lines. A2780R and TOV112D, with elevated ST3Gal1 levels, were selected for further analysis (Figure 2a). ST3Gal1 knockdown reduced VEGF‐R2 mRNA and protein expression (Figure 2b), but its overexpression had the opposite effect (Figure S1A). To verify the ST3Gal1‐VEGF‐R2 interaction, co‐immunoprecipitation was conducted by conjugating anti‐VEGF‐R2 antibody to magnetic beads, followed by immunoblotting for ST3Gal1, VEGF‐R2, and phosphorylated VEGF‐R2 (Figure 2c). In ST3Gal1‐knockdown A2780R cells, reduced ST3Gal1 and phosphorylated VEGF‐R2 levels were observed after pull‐down, supporting their interaction. We further applied sialic acid‐binding lectins, *Maackia amurensis* Lectins I & II (MAL I & II), which detect terminal α2,3‐sialylated glycans, to identify protein complexes after anti‐VEGF‐R2 antibody conjugation (Figure 2c). The downregulation of MAL I & II expression indicated that VEGF‐R2 might interact with ST3Gal1. ST3Gal1 knockdown reduced the invasive ability of cancer cells in transwell migration and invasion assays (Figure 2d,e), but overexpression showed the opposite effect (Figure S1B).

**FIGURE 2:**
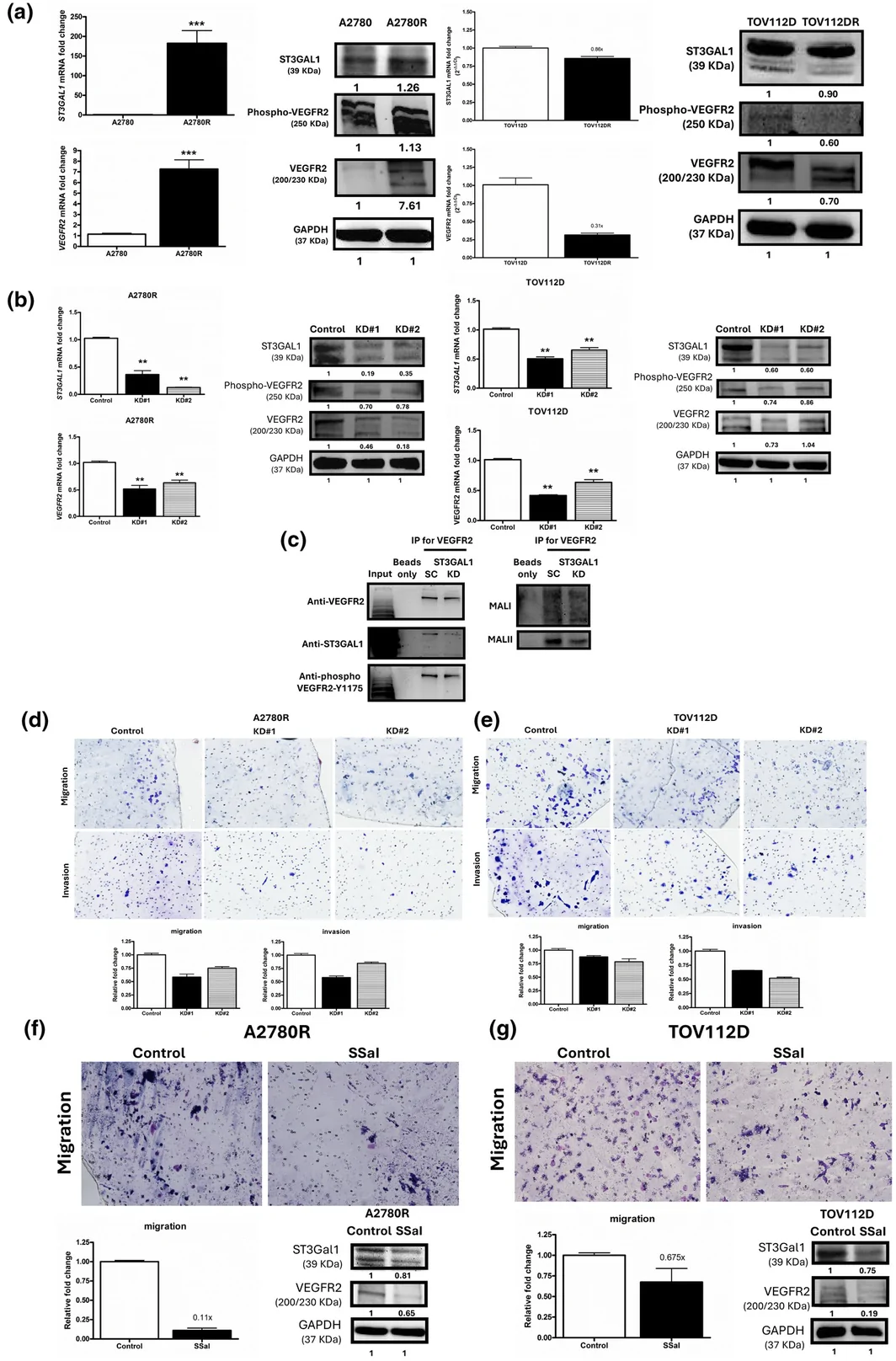
ST3Gal1 knockdown inhibited VEGF‐R2 expression and suppressed migration and invasion. (a) The expression levels of ST3Gal1 and VEGF‐R2 in four ovarian cancer (OC) cell lines (A2780, A2780R, TOV112D, and TOV112DR) were analyzed. Data represent mean ± SD ***P < 0.001. GAPDH was used as a loading control (*n* = 3 independent experiments). (b) Knockdown of ST3Gal1 in A2780R and TOV112D cells significantly reduced VEGF‐R2 mRNA and protein expression, as determined by RT‐qPCR and western blotting analysis **P < 0.01 (*n* = 3). (c) The co‐immunoprecipitation assay of ST3Gal1 and VEGF‐R2 in A2780R cells. Conjugating magnetic beads with anti‐VEGF‐R2 antibody, we detected protein complexes of ST3Gal1, VEGF‐R2, phosphorylated‐VEGF‐R2, MAL‐I and MAL‐II via immunoblotting. (d, e) Transwell migration and Matrigel invasion assays in A2780R and TOV112D transduced as indicated. Data represent mean ± SD (*n* = 3). (f, g) SsaI reduced VEGF‐R2 expression and inhibited cell migration in transwell assays (*n* = 3).

### 3.4 SsaI, a specific ST3Gal1 inhibitor, suppresses cell migration by modulating the VEGF‐R2/JAK2/STAT3 signaling pathway

SsaI exhibited potent inhibitory activity against ST3Gal1, as previously reported, leading to reduced VEGF‐R2 expression and decreased invasiveness of cancer cells in transwell migration assays (Figure 2f,g). Its effect was confirmed by reduced ST3Gal1 and VEGF‐R2 mRNA levels and transwell invasion assay results (Figure S1C).

Duolink PLA immunofluorescence localized ST3Gal1‐VEGF‐R2 interactions in A2780R cells, and the signal reduction following SsaI treatment or ST3Gal1 knockdown supported the concept that SsaI downregulates VEGF‐R2 via ST3Gal1 inhibition (Figure 3a).

**FIGURE 3:**
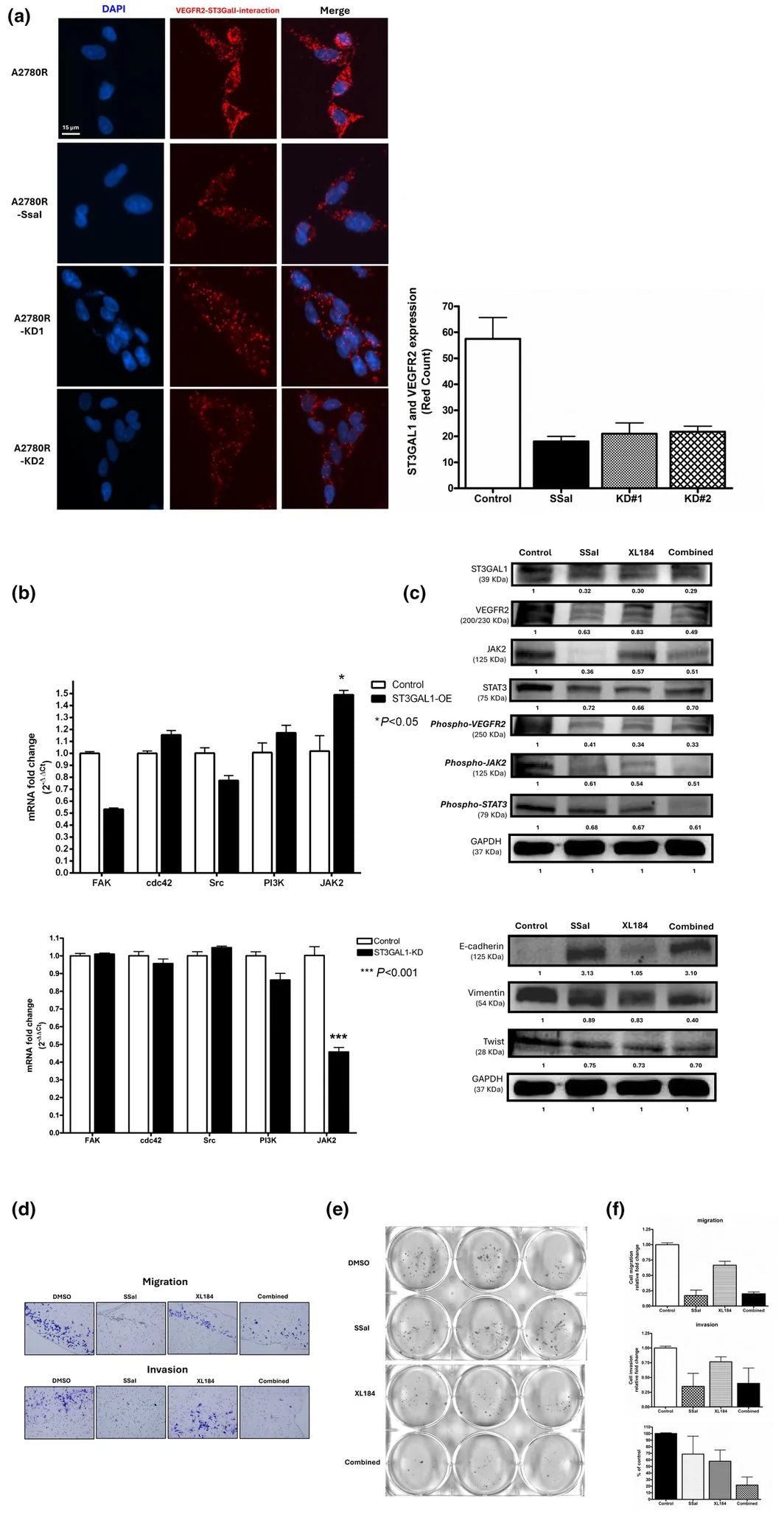
SsaI, a specific ST3Gal1 inhibitor, attenuated cell migration by modulating the VEGF‐R2/JAK2/STAT3 signaling pathway. (a) Interaction between ST3Gal1 and VEGF‐R2 in A2780R cells was detected using Duolink PLA. The signals were visualized in red and the nuclei in blue. This demonstrated that SsaI downregulated VEGF‐R2 expression by targeting ST3Gal1 (bar = 15 μm; *n* = 3). (b) RT‐qPCR analysis demonstrated that the JAK2 pathway was the most activated by ST3Gal1 *P < 0.05, ***P < 0.001 (*n* = 3). (c) Western blotting analysis of the effect of the combination of SsaI and XL184. (d–f) Combination treatment inhibited colony formation, cell migration (transwell assay), and invasion (Matrigel assay). The number of colonies was counted and plotted. Data are presented as mean ± SD (*n* = 3).

To explore pathways downstream of VEGF‐R2, RT‐qPCR was conducted under ST3Gal1 overexpression and knockdown conditions. Although the cell division cycle 42 and phosphoinositide 3‐kinase pathways were modulated by ST3Gal1, JAK2 was the most prominently activated pathway (Figure 3b).

When combined with SsaI, cabozantinib (XL184), a multi‐receptor tyrosine kinase inhibitor that binds to VEGF‐R2, further suppressed the ST3Gal1/VEGF‐R2/JAK2/STAT3 axis, especially its phosphorylated forms (Figure 3c). This combination also reduced the epithelial‐mesenchymal transition (Figure 3c) and impaired A2780R cell colony formation, migration, and invasion (Figure 3d–f).

### 3.5 SsaI and XL184 combination suppresses tumor formation in vivo

To validate these findings in vivo, A2780R cells were injected into the flanks of nude mice, which were treated accordingly (Figure 4a). Average tumor size was markedly reduced in the combination group (Figure 4b), whereas average tumor volume and weight were significantly lower with combination treatment (Figure 4c). However, no significant differences were observed between the combination and XL184 groups. Immunoblotting showed enhanced downregulation of the ST3Gal1/VEGF‐R2/JAK2/STAT3 axis and epithelial–mesenchymal transition markers with combination treatment (Figure 4d). Immunostaining revealed decreased expressions of ST3Gal1, VEGF‐R2, JAK2, STAT3, Ki‐67, and Duolink PLA in the combination group (Figure 4e,f). The effects of SsaI and XL184 are shown in Figure S2. These findings indicated that combined treatment with SsaI and XL184 suppressed ST3Gal1/VEGF‐R2/JAK2/STAT3 signaling and tumor growth in vivo, although the reduction in tumor burden was not significantly different from that of XL184 treatment alone.

**FIGURE 4:**
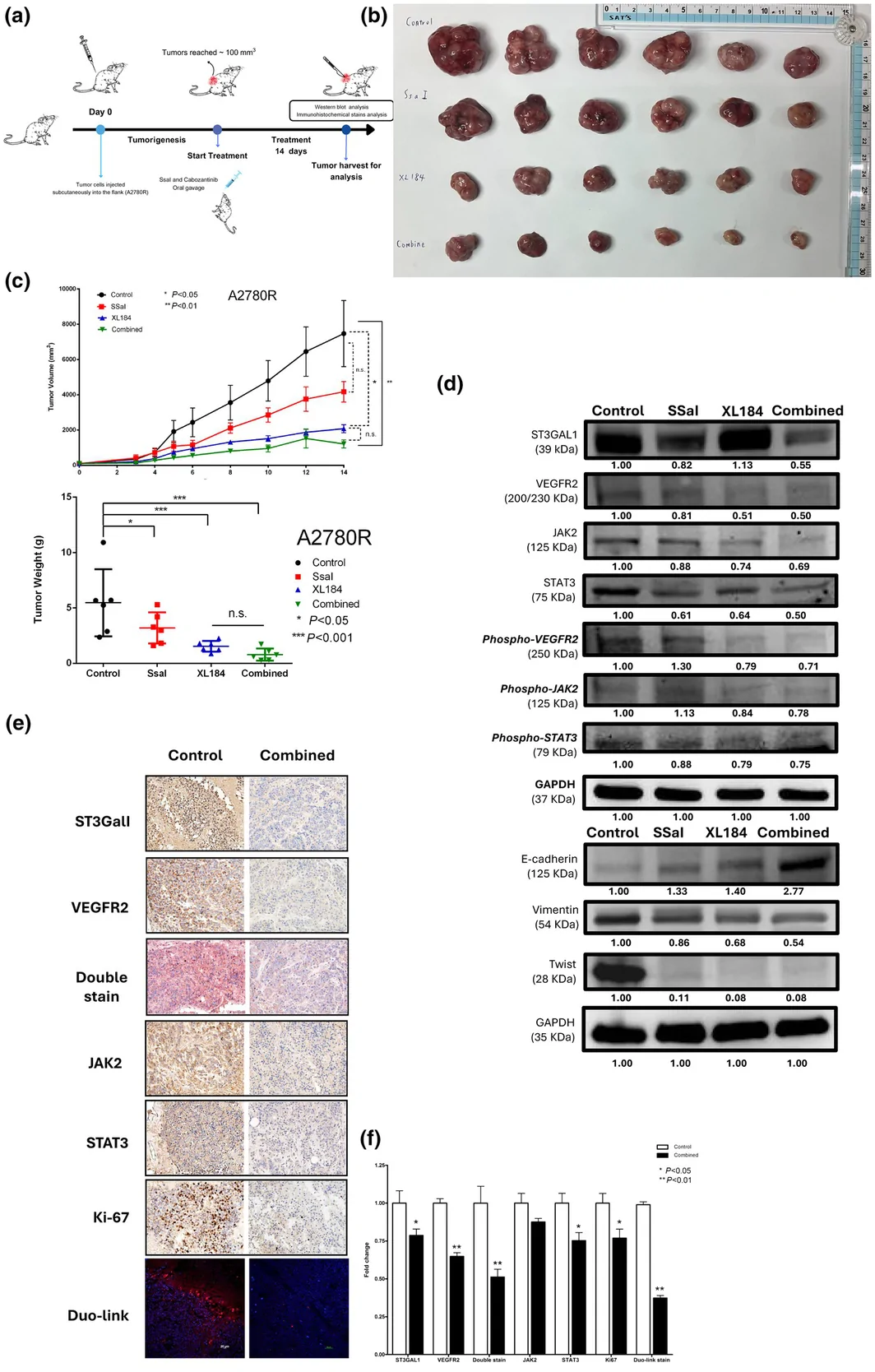
SsaI and XL184 combination inhibits tumor growth in vivo. (a) Schematic representation of the xenograft study. Created using Canva. (b, c) In vivo effects of SsaI and XL184 combination on tumor growth in xenografts. A2780R cells were subcutaneously injected into the dorsal flanks of nude mice. Representative tumor images and tumor weights were recorded. The tumor volumes were measured every 3 days. Data represent mean ± SD (*n* = 6). (d) Western blotting analysis of the effect of combining SsaI and XL184 in vivo. (e, f) This image shows IHC staining for ST3Gal1, VEGF‐R2, JAK2, STAT3, Ki‐67, and Duolink PLA in the xenograft tumor tissue. Data represent mean ± SD (*n* = 3).

## 4 DISCUSSION

This study suggests that the ST3Gal1 pathway may be involved in tumor progression in ovarian cancer models (E‐OC as an example in the current study), potentially through modulation of the VEGF‐R2/JAK2/STAT3 signaling pathway (Figure 5). Targeting ST3Gal1 with SsaI, especially combined with VEGF‐R2 inhibitor XL184, effectively suppressed tumor growth and invasion in vitro and in vivo. To clarify the overall study framework, we have added a graphical schematic summarizing the translational workflow of the study (Figure S3).

**FIGURE 5:**
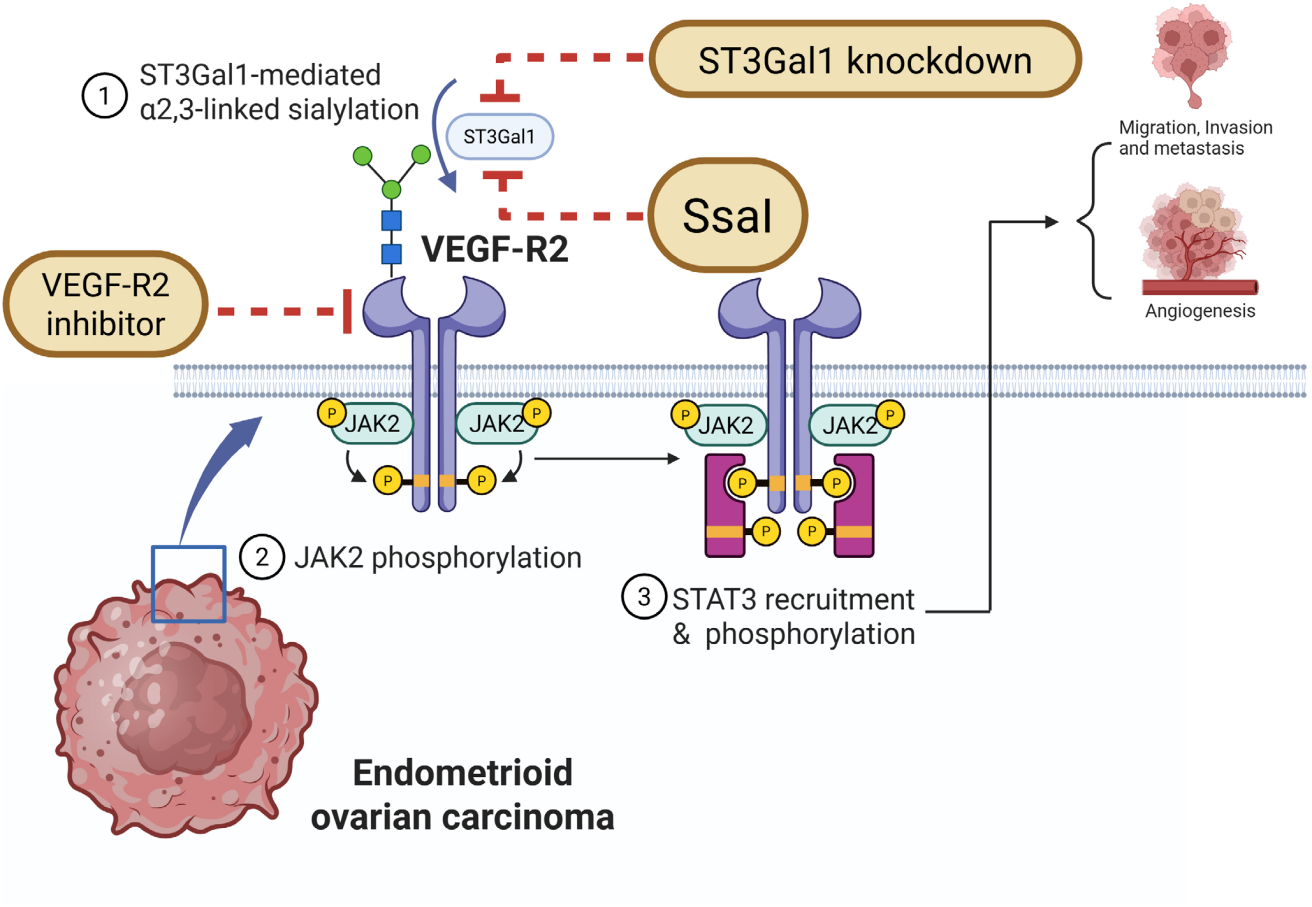
Proposed schema illustrating the role of ST3Gal1 in modulating the VEGF‐R2/JAK2/STAT3 signaling pathway in endometrioid‐type ovarian cancer (E‐OC). Created using BioRender.com.

Aberrant glycosylation, particularly sialylation, is a key post‐translational modification associated with various diseases.^18^, ^19^, ^20^, ^21^ Sialylation, catalyzed by sialyltransferases (STs), adds sialic acids to the terminal ends of glycans.^22^ Based on the attachment site, STs are classified into four subtypes: ST3Gal (ST3Gal1–6), ST6Gal (1 and 2), ST6GalNAc (six members), and ST8Sia (six members), with ST3 and ST6 being the most studied in OC.^12^, ^13^, ^18^, ^22^, ^23^, ^24^ Our previous study showed that ST3Gal1 was highly expressed in high‐grade serous OC and clear‐cell OC,^12^, ^13^, ^24^ but the present study demonstrated elevated ST3Gal1 expression in E‐OC, where it regulated cell migration.

VEGF and its receptors are central to angiogenesis. Bevacizumab, the first approved VEGF‐A‐targeting monoclonal antibody, inhibits VEGF‐A/VEGF‐R2 signaling and tumor angiogenesis.^25^, ^26^ In the first‐line maintenance therapy setting, as demonstrated by the GOG‐0218^27^ and ICON 7^28^ trials, bevacizumab demonstrated a significant PFS benefit but only a numerical OS improvement in patients with ads‐OC (not statistically significant). In the PAOLA‐1 trial,^29^, ^30^ maintenance therapy with a PARP inhibitor (olaparib) plus bevacizumab significantly improved both PFS and OS in patients with ads‐OC homologous recombination deficiency‐positive disease, especially those with *BRCA*m. However, patients with homologous recombination deficiency‐positive disease but without *BRCA*m showed a significant PFS benefit but only numerical OS improvement. In recurrent OC, bevacizumab showed a significant PFS benefit but no significant OS improvement across both platinum‐resistant recurrent OC (AURELIA and JGOG 3023 trials) and platinum‐sensitive recurrent OC (OCEANS, GOG‐0213 and MITO16 trials) settings.^11^, ^31^, ^32^, ^33^, ^34^, ^35^

Real‐world data on bevacizumab maintenance therapy in OC show significant improvement in PFS but not OS, highlighting the need to enhance the efficacy and overcome resistance to anti‐angiogenic therapy. Our previous research showed that ST3Gal1 is overexpressed in ads‐EC and associated with a poor prognosis.^14^ ST3Gal1 directly glycosylates VEGF‐A, sustaining its expression and downstream signaling.^14^ SsaI, a potent and selective ST3Gal1 inhibitor,^36^, ^37^, ^38^ may overcome anti‐VEGF‐A resistance and enhance bevacizumab efficacy by targeting ST3Gal1. Our present study also showed that ST3Gal1 altered α2,3‐linked sialylation of VEGF‐R2 and was associated with a poor prognosis in E‐OC. By modulating angiogenesis through VEGF‐R2 signaling, ST3Gal1 represents a novel anti‐angiogenic target in OC. These findings support its potential as a therapeutic target to overcome anti‐VEGF‐R2 resistance in ads‐OC. Notably, angiogenesis plays an important role in chemoresistance development in OC.^11^, ^35^ Therefore, both parental and platinum‐resistant OC cell lines were initially evaluated in this study, as resistant tumor phenotypes are frequently associated with enhanced pro‐angiogenic signaling that may contribute to tumor survival and therapeutic resistance.^2^, ^39^

Several study limitations should be acknowledged. First, the clinical cohort was relatively small (*n* = 53), with only 21 cases in the high ST3Gal1 expression group, which may have limited the statistical power and subsequently over‐ or underestimated the parameters. This limitation can be clearly reflected by the absence of an association between CRS completeness and outcomes among patients with E‐OC in the univariable analysis (Table 1). As evidence is strong enough to approve R0 after CRS as a very important independent favorable prognostic factor while evaluating the outcomes of patients with OC,^4^, ^5^, ^16^, ^40^ the lack of statistical significance of CRS completeness to prognosis but the presence of statistical significance between ST3Gal1 expression and prognosis in the univariable analysis hints that ST3Gal1 expression may be associated with outcomes of patients with E‐OC. Of course, the absence of the role of CRS completeness on the impact of prognosis may be influenced by the FIGO stage. In fact, the strong association between FIGO stage and the outcomes of patients with E‐OC in the univariable and multivariable analyses suggests that the independent prognostic value of ST3Gal1 expression on outcomes of patients with E‐OC may be also influenced by FIGO stage. Importantly, ST3Gal1 did not retain statistical significance in the multivariable analysis and therefore cannot be considered an independent prognostic factor in the current study.

Second, unlike the high proportion of high‐grade serous OC cases in the OC cohort, E‐OC comprised only a small proportion of the entire OC cohort (*n* = 25 to *n* = 62) (Figure 1a,b) in the large well‐known public tissue banks such as The Cancer Genome Atlas, Oncomine (19.1%; 25/131) and Kaplan–Meier Plotter databases (KM plotter, 4.8%; 62/1294). Although the current study enrolled 53 E‐OC patients, it may not be considered a small series. However, because of this uncontrolled limitation, the use of public datasets (Oncomine or KM plotter) to validate the role of ST3Gal1 on prognosis of E‐OC must be interpreted with caution. To overcome the limitation secondary to a small sample size, an independent validation in larger, subtype‐specific datasets is warranted. Therefore, the similar finding of the current study, which included only 53 patients with E‐OC, faced a similar challenge. These factors may have resulted in our failure to demonstrate the statistical significance of ST3Gal1 on the outcomes of patients with E‐OC in the multivariable analysis.

Third, no other molecular classifications (e.g. DNA polymerase epsilon [*POLE*] mutation [ultramutated], mismatch repair (*MMR*) status, microsatellite instability [*MSI*], copy‐number status, p53‐abnormal, and no specific molecule profile [NSMP]) were routinely evaluated in this retrospective cohort study, although they may become of interest in the management of patients with OC,^15^, ^41^, ^42^ particularly those with endometriosis‐associated OC.^15^ In fact, the aforementioned and cross‐century‐developed molecular system is playing a critical and determinative role and is importantly essential to the establishment of a new FIGO 2023 endometrial cancer staging system, which not only provides highly accurate outcome predictions but also suggests the efficacy of biomarker‐guided agonistic treatment.^42^, ^43^, ^44^ However, in terms of gynecologic cancers, endometrial cancer may be the only well‐accepted cancer suitable for the aforementioned molecular classification; in contrast, for OC, *BRCA* mutation and homologous recombination status, regardless of classified OC subtypes, may be much more important, particularly while PARP inhibitors have become well‐accepted in the maintenance therapy for ads‐OC after primary treatment.^3^, ^8^, ^9^ Given increasing evidence that genomic subtypes significantly influence prognosis and therapeutic response in OC, the prognostic relevance of ST3Gal1 requires validation in genomically characterized cohorts, particularly for those patients with E‐OC or another endometriosis‐associated OC, such as the clear cell carcinoma subtype, as the recent trend for managing OC is focusing primarily on patients with high‐grade serous OC.

Fourth, although our data suggest an association between ST3Gal1 and VEGF‐R2 and indicate that ST3Gal1 modulation may influence VEGF‐R2 signaling, the precise molecular or functional mechanisms remain to be fully elucidated. In particular, the specific ST3Gal1‐modified glycosylation sites on VEGF‐R2 were not identified in the present study. Moreover, whether altered α2,3‐sialylation directly affects VEGF‐R2 activation, receptor trafficking, or protein stability requires further investigation using site‐specific mutagenesis or glycoproteomic approaches. This basic research requires a highly time‐consuming procedure, so our future work will explore this idea.

Fifth, although this study focused on E‐OC, direct comparative analyses across different OC histologic subtypes were not performed. Therefore, whether ST3Gal1‐mediated regulation of the VEGF‐R2/JAK2/STAT3 signaling axis is specific to E‐OC remains to be determined and warrants further investigation.

Sixth, the translational feasibility of SsaI remains limited, although many articles have explored its competitive ability with ST3Gal1.^12^, ^13^, ^14^, ^36^, ^37^, ^38^, ^45^, ^46^, ^47^ SsaI is a selective inhibitor of STs through biochemical screening of natural products.^36^, ^37^, ^38^ Wu et al.^36^ demonstrated that SsaI acts as a competitive inhibitor of ST3Gal1 and exhibits selectivity without affecting other glycosyltransferases or glycosidases, establishing its enzymatic selectivity and inhibitory kinetics. Subsequent studies reported biologic activities of SsaI in experimental models, including anti‐inflammatory effects in trinitrobenzene sulfonic acid‐induced colitis, improved inflammatory and metabolic parameters in metabolic disease models, and modulated glycosylation‐related cellular processes.^45^, ^46^, ^47^ However, a comprehensive evaluation of pharmacokinetics, systemic toxicity, bioavailability, and clinical feasibility remains lacking. Further pharmacologic and translational studies are required to clarify the therapeutic potential of ST3Gal1 inhibitors in clinical settings.

Seventh, the in vivo experiments in this study were performed using a subcutaneous xenograft model. Although this model allows reproducible tumor growth and convenient monitoring of tumor volume, it may not fully replicate the organ‐specific tumor microenvironment or metastatic behavior of OC. Orthotopic tumor models reportedly better mimic tumor‐host interactions, invasion, and metastatic dissemination because tumor cells grow within their native organ environment.^48^ In contrast, subcutaneous models provide practical advantages for tumor measurement and therapeutic evaluation but may underestimate metastatic potential. Future studies using orthotopic OC models may further clarify the role of ST3Gal1 in tumor progression and metastasis.

Finally, immunohistochemistry scoring was performed by a single pathologist, and interobserver reproducibility was not assessed, which may introduce potential observer bias.

In conclusion, although ST3Gal1 was associated with adverse outcomes in univariable analysis, it failed to demonstrate independent prognostic significance in multivariable analysis. As anti‐angiogenic agents have become attractive backbone treatment strategies in the management of OC, many challenges and uncertainties of ST3Gal1 on E‐OC outcomes require further validation regardless of subtype classification. However, based on these findings, we propose that the ST3Gal1 pathway may interact with VEGF‐R2 signaling and metastatic behavior in ovarian cancer models (E‐OC as an example in the current study). Both in vitro and in vivo experiments demonstrated that ST3Gal1 modulation attenuates VEGF‐R2 signaling and suppresses tumor cell migration, invasion, and tumor growth. Combined inhibition of ST3Gal1 and VEGF‐R2 reduced tumor growth in preclinical models, but further clinical validation is required before identification of its clinical relevance.

## AUTHOR CONTRIBUTIONS

Wei‐Ting Chao contributed to conception and design of study, data collection, analysis and interpretation, statistical analysis, manuscript preparation, and patient recruitment. Chia‐Hao Liu contributed to conception and design of study, and data analysis and interpretation. Szu‐Ting Yang and Liang‐Wei Wang contributed to manuscript preparation. Chen‐Hao Lin contributed to conception and design of study, data analysis and interpretation, and statistical analysis. Peng‐Hui Wang contributed to conception and design of study, data analysis and interpretation, and manuscript preparation. W‐TC and C‐HL were responsible for imaging.

## FUNDING INFORMATION

This research was supported by grants from the Taipei Veterans General Hospital (V114C‐039, V114B‐015, V115C‐095, and V115B‐008) and the Taiwan National Science and Technology Council, Executive Yuan (110‐2314‐B‐075‐016 MY3 and 113‐2314‐B‐075‐057‐MY3), Taipei, Taiwan. The authors appreciate the support from the Taiwan Female Cancer Foundation (IJG‐D‐26‐00253), Taipei, Taiwan.

## CONFLICT OF INTEREST STATEMENT

The authors have no conflicts of interest.

## Supporting information


**Figure S1.** (A) Overexpression of ST3GalI in A2780 and TOV112DR cells resulted in a significant increase in the mRNA and protein expression levels of VEGFR2, as determined by qRT‐PCR and Western blot analysis (*n* = 3). (B) Overexpression of ST3GalI resulted in an upregulation of cell migration and invasion in vitro assays (*n* = 3). (C) SsaI reduced mRNA expression of ST3GalI and VEGFR2 and inhibited transwell invasion assay (*n* = 3).

**Figure S2.** Immunohistochemical staining for ST3GalI, VEGFR2, JAK2, STAT3, Ki‐67, and Duolink PLA in the xenograft tumor tissue.

**Figure S3.** ST3Gal1 regulates endometrioid‐type epithelial ovarian cancer cell migration and invasion via VEGF‐R2/JAK2/STAT3 signaling cascades.

**Appendix S1.** Human cell‐line authentication report.

## Data Availability

All data analyzed during this study are available upon reasonable request.
