# Supplementary material for: α2,3‐Sialyltransferase (ST3Gal1) regulates endometrioid‐type epithelial ovarian cancer cell migration and invasion via VEGF‐R2/JAK2/STAT3 signaling cascades
Source: Int J Gynaecol Obstet. 2026 May 4;175(1):453–65. doi: 10.1002/ijgo.71057 (PMC13629583; doi:10.1002/ijgo.71057)

細胞鑑定檢測報告書

委託單位：台北榮民總醫院 婦女醫學部

委託者：王鵬惠

電話：02-2871-2121#82218

地址：台北市北投區石牌路二段 322 號致德樓 5 樓 R527 室

應用技術實驗室

電話：02-26961658

地址：新北市汐止區新台五路一段 92 號 4 樓

網址：www.genomics.com.tw

Human Cell Line Authentication Report

Name of institution	台北榮民總醫院 婦女醫學部	Receive Date	2025.05.16
Principal investigator	王鵬惠	Report Date	2025.05.29
Original Material	■Human Cell Line ▫Human gDNA		
Analysis Method	▫Comparison between samples ■Comparison with database		
Sample Name	A2780		
Note			

1 Workflow:

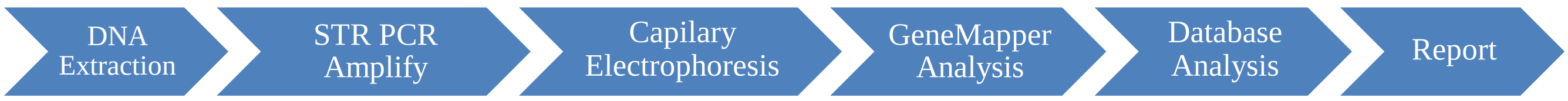

2 Method:

2.1 DNA Extraction：Use DNeasy Blood & Tissue Kit（QIAGEN, 69504）to perform DNA extraction and purification. The detailed operation procedure refers to the kit's User Manual.

2.2 Sample，together with positive and negative control were amplified using AmpFLSTR™ Identifiler™ Plus PCR Amplification Kit (ThermoFisher, 4427368)。The detailed operation procedure refers to the kit's User Manual.

2.3 Amplified products were processed using the ABI3730xl Genetic Analyzer .

2.4 Data were analyzed using GeneMapper4.0 software and then compared with the **DSMZ** database。

3 Result:

3.1 Table 1. Matching information on the cell lines

NO	Cell line matched	Source	Similarity
1	A2780	HPACC	100 %
2	A2780cis	HPACC	93.8 %
3	A2780ADR	HPACC	90.6 %

3.2 Figure 1. STR matching analysis

Similarity	Cell line	Source	Shared	D5S818	D7S820	D13S317	D16S539	vWA	TH01	TPOX	CSF1PO	Amelogenin	D3S1358	D21S11	D18S51	D8S1179	FGA	D19S433	D2S1338
		Your query		11, 12	10, 10	12, 13	11, 13	15, 16	6, 6	8, 10	10, 11	X, X	14, 16	28, 28	16, 18	15, 17	19, 24	12, 12	21, 22
100 %	A2780	HPACC	16	11, 12	10, 10	12, 13	11, 13	15, 16	6, 6	8, 10	10, 11	X, X	14, 16	28, 28	16, 18	15, 17	19, 24	12, 12	21, 22
93.8 %	A2780cis	HPACC	16	11, 11	10, 10	13, 13	11, 13	15, 16	6, 6	8, 10	10, 11	X, X	14, 16	28, 28	16, 18	15, 17	19, 24	12, 12	21, 22
90.6 %	A2780ADR	HPACC	16	11, 12	10, 10	13, 13	11, 14	15, 16	6, 6	8, 9	10, 11	X, X	14, 16	28, 28	16, 18	15, 17	19, 24	12, 12	21, 22

886-2-2696-1658 886-2-2696-1589 www.genomics.com.tw 22102 新北市汐止區新台五路一段100號14樓

3.2.1 Note:

3.2.1.1 The human STR profile database includes data sets of 2455 cell lines from ATCC, DSMZ, JCRB and RIKEN.

3.2.1.2 Based on the ANSI Standard, cell lines with≧80% match are considered to be related. Cell lines with between a 55% to 80% match require further profiling for authentication of relatedness.

3.2.1.3 The similarity score is calculated:

$$\frac{2 \cdot A_{shared}}{A_{query} + A_{ref}}$$

3.3 Table 2. 16 Loci STR Profile

Loci	STR Profile	
	**Allele1**	**Allele2**
D8S1179	15	17
D21S11	28	28
D7S820	10	10
CSF1PO	10	11
D3S1358	14	16
TH01	6	6
D13S317	12	13
D16S539	11	13
D2S1338	21	22
D19S433	12	12
vWA	15	16
TPOX	8	10
D18S51	16	18
AMEL	X	X
D5S818	11	12
FGA	19	24

Operating Staff /Date:蔡佳穎 2025.05.29 Lab Manager/Date: 陳素蓮 2025.05.29

Applied Biosystems
GeneMapper 4.0

2025.05.28 STR

Sample Name	Panel	SQI	SQ
A2780	Identifiler_v1		

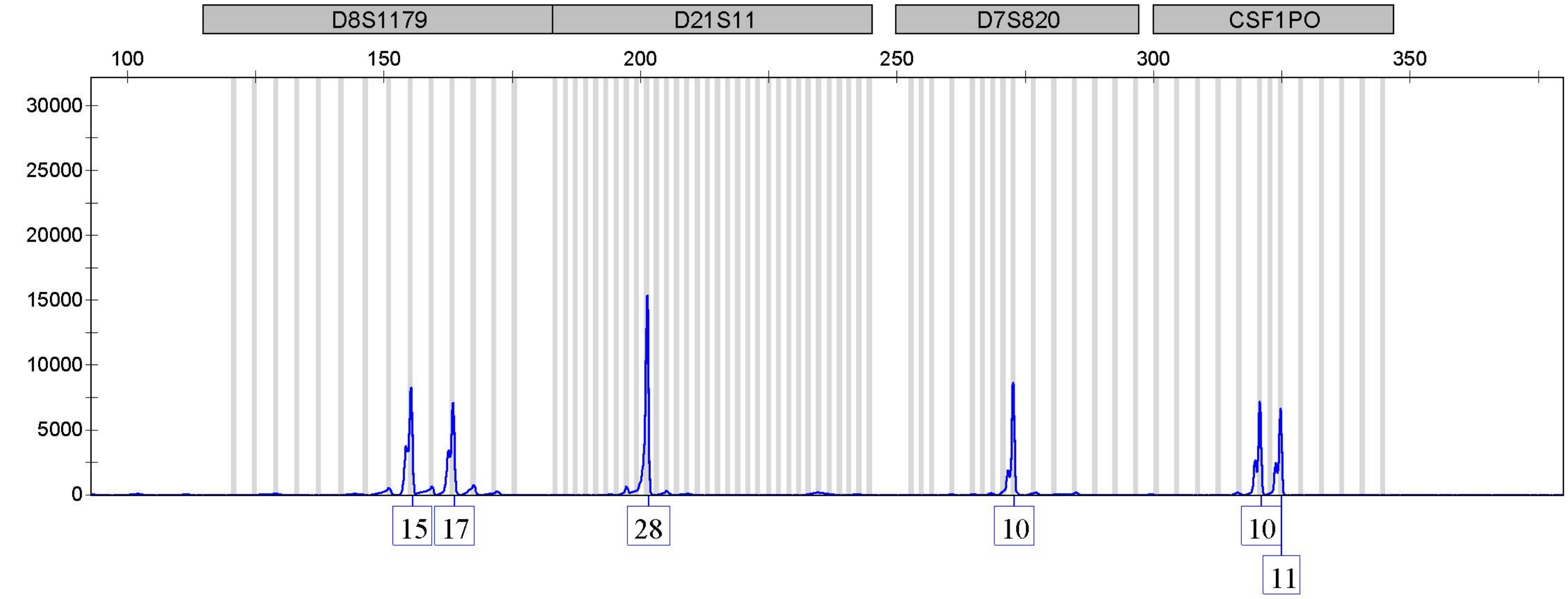

A2780	Identifiler_v1		

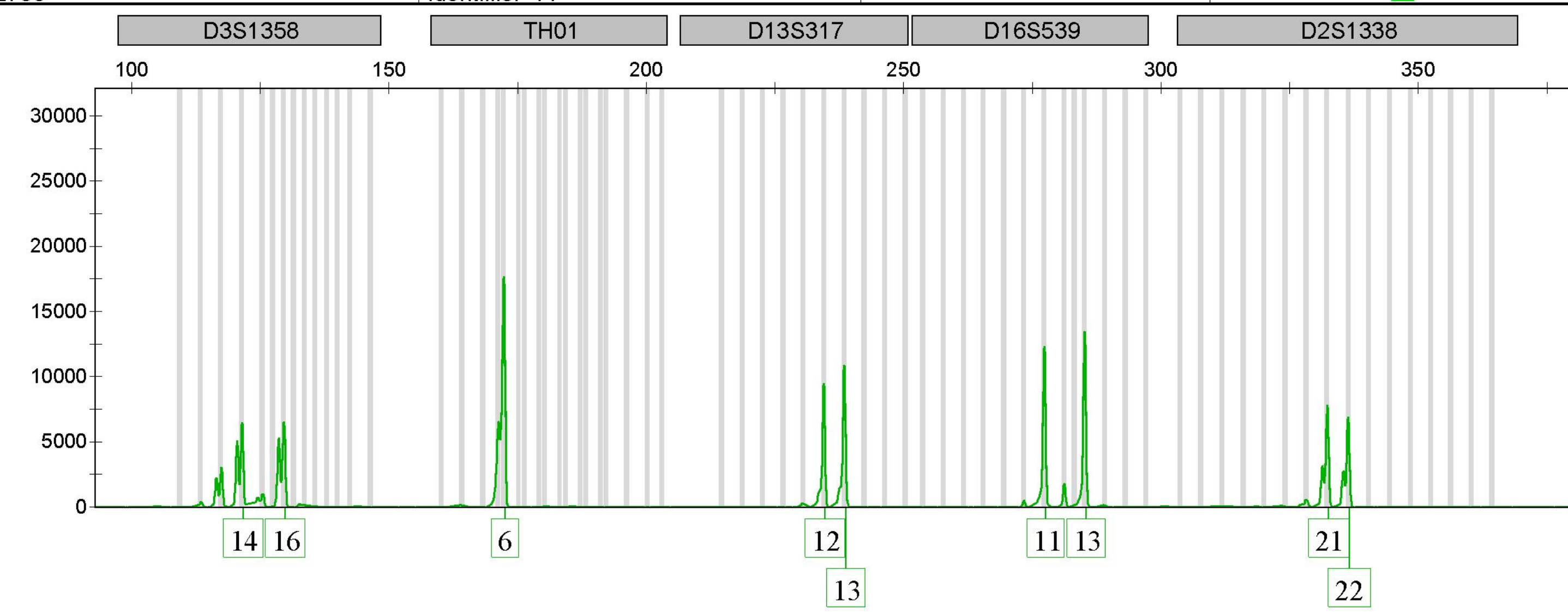

Applied Biosystems

GeneMapper 4.0

2025.05.28 STR

Sample Name	Panel	SQI	SQ
A2780	Identifiler_v1		

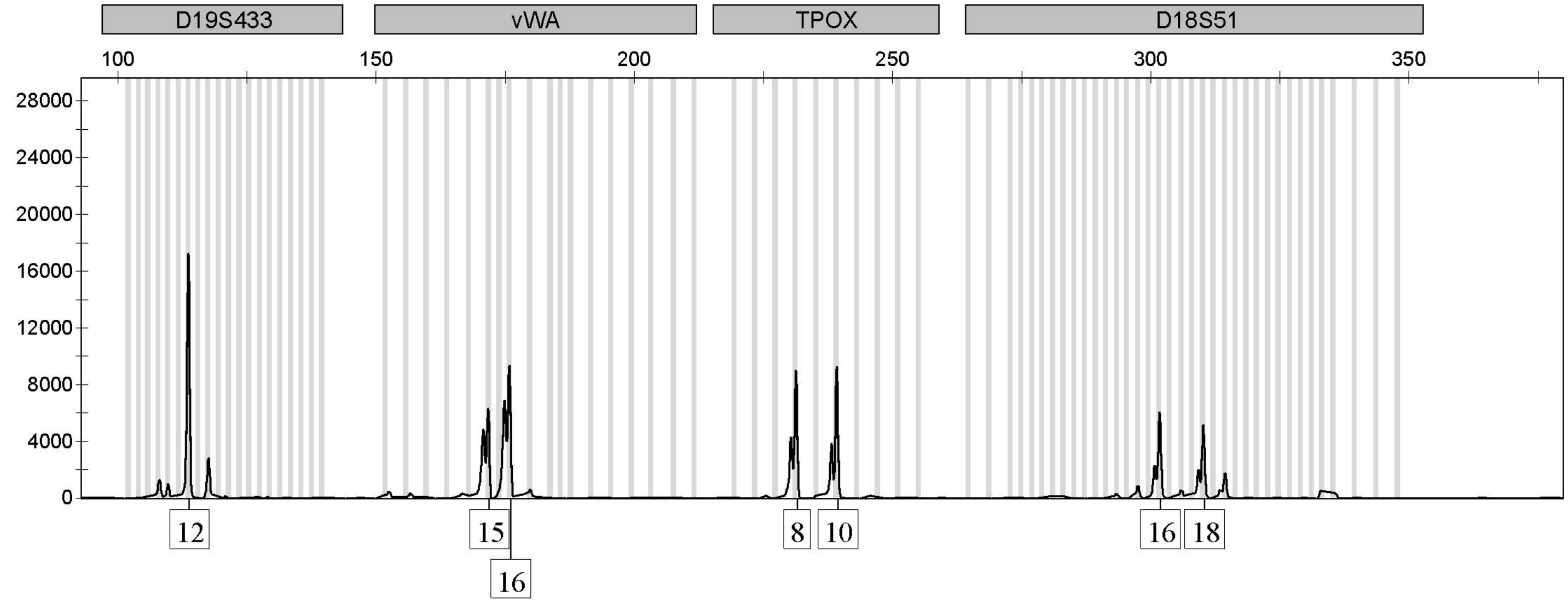

A2780	Identifiler_v1		

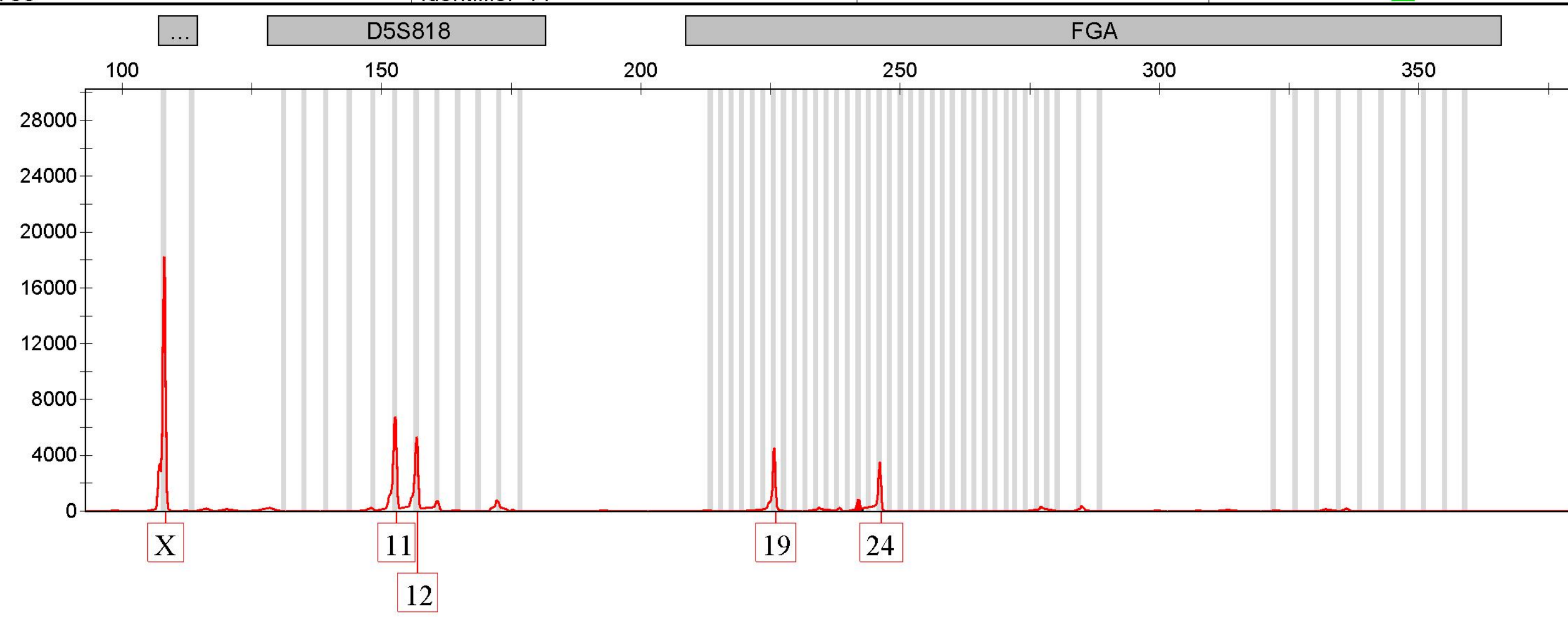

細胞鑑定檢測報告書

委託單位：台北榮民總醫院 婦女醫學部

委託者：王鵬惠

電話：02-2871-2121#82218

地址：台北市北投區石牌路二段 322 號致德樓 5 樓 R527 室

應用技術實驗室

電話：02-26961658

地址：新北市汐止區新台五路一段 92 號 4 樓

網址：www.genomics.com.tw

Human Cell Line Authentication Report

Name of institution	台北榮民總醫院 婦女醫學部	Receive Date	2025.05.16
Principal investigator	王鵬惠	Report Date	2025.05.29
Original Material	■Human Cell Line □Human gDNA		
Analysis Method	□Comparison between samples ■Comparison with database		
Sample Name	TOV112D		
Note			

1 Workflow:

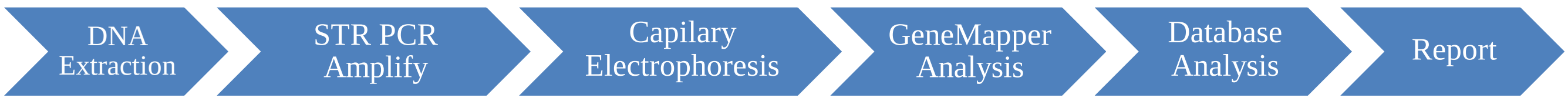

2 Method:

2.1 DNA Extraction：Use DNeasy Blood & Tissue Kit（QIAGEN, 69504）to perform DNA extraction and purification. The detailed operation procedure refers to the kit's User Manual.

2.2 Sample，together with positive and negative control were amplified using AmpFLSTR™ Identifiler™ Plus PCR Amplification Kit (ThermoFisher, 4427368)。The detailed operation procedure refers to the kit's User Manual.

2.3 Amplified products were processed using the ABI3730xl Genetic Analyzer .

2.4 Data were analyzed using GeneMapper4.0 software and then compared with the **DSMZ** database。

3 Result：

3.1 Table 1. Matching information on the cell lines

NO	Cell line matched	Source	Similarity
1	TOV-112D	CRL-11731	100 %

3.2 Figure 1. STR matching analysis

Similarity	Cell line	Source	Shared	D5S818	D7S820	D13S317	D16S539	vWA	TH01	TPOX	CSF1PO	Amelogenin	D3S1358	D21S11	D18S51	D8S1179	FGA	D19S433	D2S1338
		Your query		10, 10	9, 10	8, 8	9, 12	18, 18	6, 6	8, 11	12, 12	X, X	15, 15	31, 31	17, 17	9, 13	20, 20	14, 14	19, 24
100 %	TOV-112D	CRL-11731	16	10, 10	9, 10	8, 8	9, 12	18, 18	6, 6	8, 11	12, 12	X, X	15, 15	31, 31	17, 17	9, 13	20, 20	14, 14	19, 24

886-2-2696-1658 886-2-2696-1589 www.genomics.com.tw 22102 新北市汐止區新台五路一段100號14樓

3.2.1 Note:

3.2.1.1 The human STR profile database includes data sets of 2455 cell lines from ATCC, DSMZ, JCRB and RIKEN.

3.2.1.2 Based on the ANSI Standard, cell lines with≧80% match are considered to be related. Cell lines with between a 55% to 80% match require further profiling for authentication of relatedness.

3.2.1.3 The similarity score is calculated:

$$\frac{2 \cdot A_{shared}}{A_{query} + A_{ref}}$$

3.3 Table 2. 16 Loci STR Profile

**Loci**	**STR Profile**	
	**Allele1**	**Allele2**
D8S1179	9	13
D21S11	31	31
D7S820	9	10
CSF1PO	12	12
D3S1358	15	15
TH01	6	6
D13S317	8	8
D16S539	9	12
D2S1338	19	24
D19S433	14	14
vWA	18	18
TPOX	8	11
D18S51	17	17
AMEL	X	X
D5S818	10	10
FGA	20	20

Operating Staff /Date:蔡佳穎 2025.05.29 Lab Manager/Date: 陳素蓮 2025.05.29

Sample Name	Panel	SQI	SQ
TOV112D	Identifiler_v1		

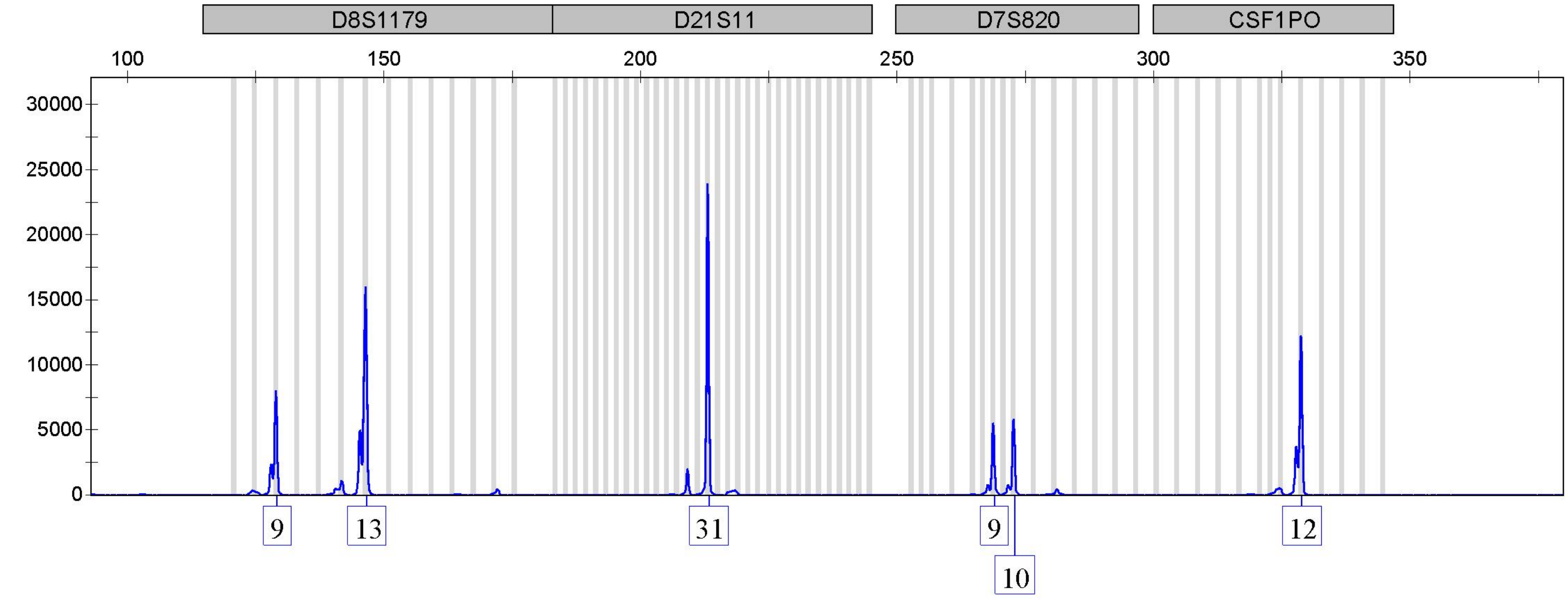

TOV112D	Identifiler_v1		

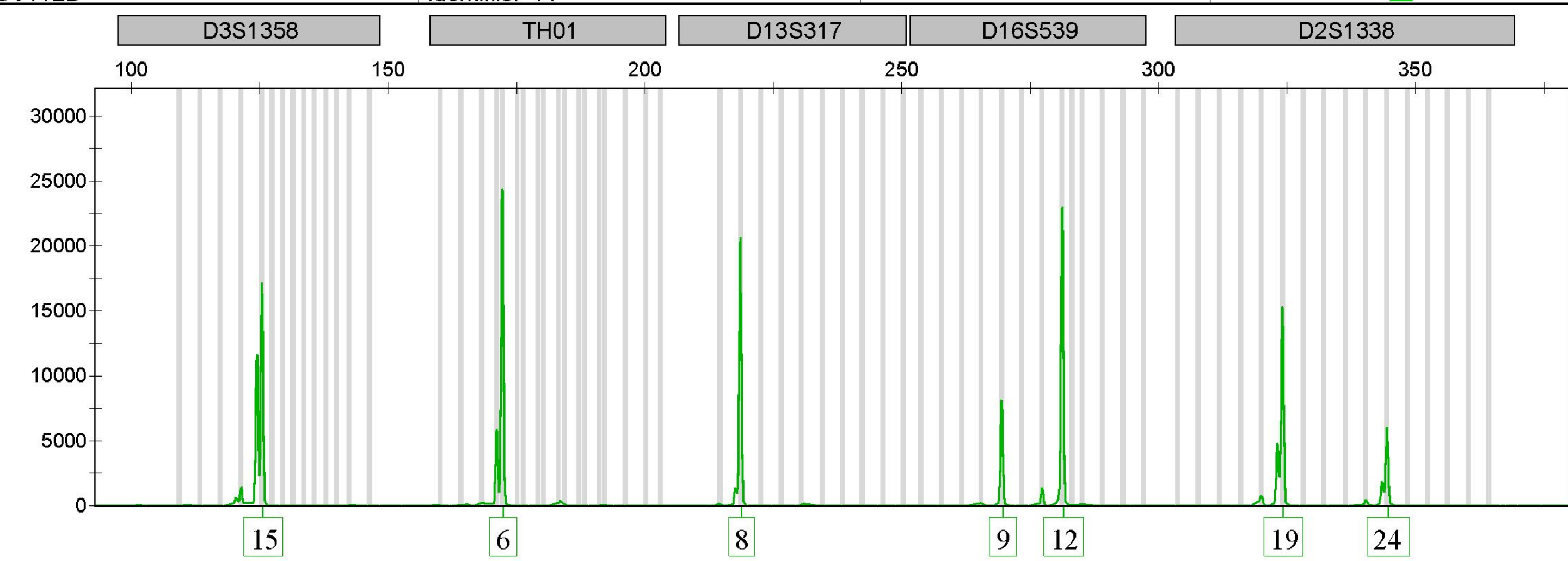

Sample Name	Panel	SQI	SQ
TOV112D	Identifiler_v1		

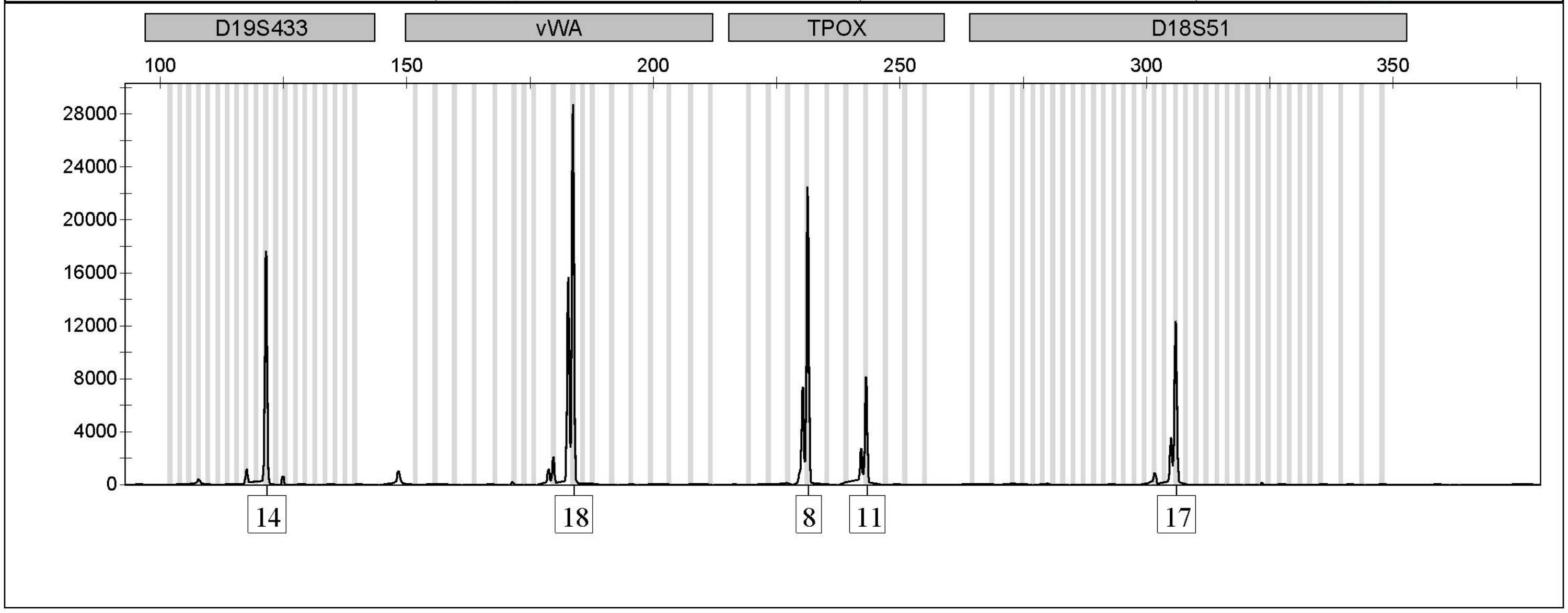

TOV112D	Identifiler_v1		

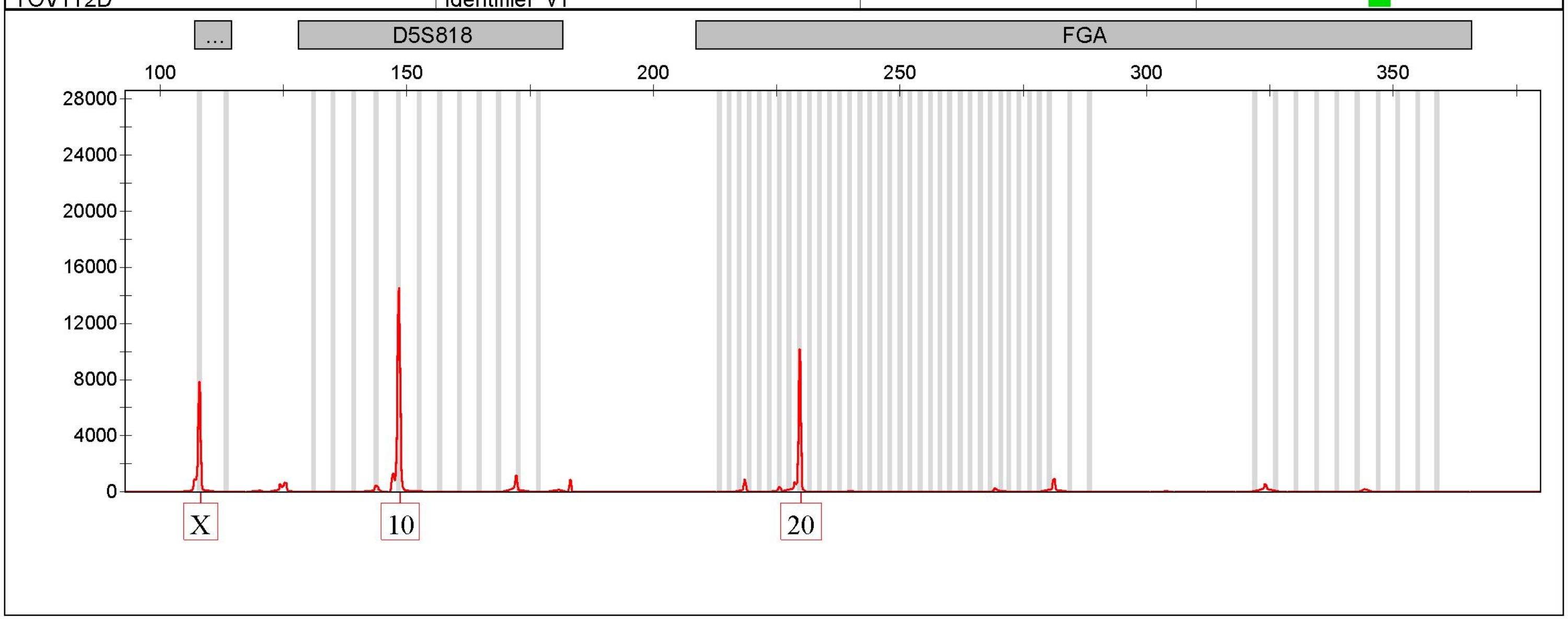

Supplement: Supplementary file 1 [file IJGO-175-453-s004.pdf]
