# Supplementary material for: α2,3‐Sialyltransferase (ST3Gal1) regulates endometrioid‐type epithelial ovarian cancer cell migration and invasion via VEGF‐R2/JAK2/STAT3 signaling cascades
Source: Int J Gynaecol Obstet. 2026 May 4;175(1):453–65. doi: 10.1002/ijgo.71057 (PMC13629583; doi:10.1002/ijgo.71057)
Supplement: Supplementary file 2 [file IJGO-175-453-s001.docx]

**Supplementary figure legends :**

**Fig.S1** (A) The overexpression of ST3GalI in A2780 and TOV112DR cells resulted in a significant increase in the mRNA and protein expression levels of VEGFR2, as determined by qRT-PCR and Western blot analysis (n = 3). (B) The overexpression of ST3GalI resulted in an upregulation of cell migration and invasion in vitro assays (n=3). (C) SsaI reduced mRNA expression of ST3GalI and VEGFR2 and inhibited transwell invasion assay (n =3).

A

**TOV112DR**

**TOV112DR**

**A2780**

**TOV112DR**

B

C
