# Supplementary material for: α2,3‐Sialyltransferase (ST3Gal1) regulates endometrioid‐type epithelial ovarian cancer cell migration and invasion via VEGF‐R2/JAK2/STAT3 signaling cascades
Source: Int J Gynaecol Obstet. 2026 May 4;175(1):453–65. doi: 10.1002/ijgo.71057 (PMC13629583; doi:10.1002/ijgo.71057)
Supplement: Supplementary file 3 [file IJGO-175-453-s003.docx]

**Supplementary figure legends :**

**Fig.S2** (A) This image showed IHC staining for ST3GalI, VEGFR2, JAK2, STAT3, Ki-67, and Duolink PLA in the xenograft tumor tissue

A
